# A predictive model for long-term coronary artery lesion risk in Kawasaki disease

**DOI:** 10.3389/fped.2026.1837865

**Published:** 2026-05-28

**Authors:** Qianzi Ge, Hongmei Chen, Shuhui Wang, Mimi Lu, Wenhui Qin, Weiguo Qian

**Affiliations:** 1 Department of Cardiology, Children’s Hospital of Soochow University, Suzhou, China; 2 Department of Emergency, Children’s Hospital of Soochow University, Suzhou, China

**Keywords:** coronary artery lesion, Kawasaki disease, logistic regression, nomogram model, risk factors

## Abstract

**Introduction:**

A major complication of Kawasaki disease (KD) is coronary artery lesions (CALs), which can lead to myocardial ischemia, myocardial infarction, and even mortality. Therefore, identifying risk factors for CALs is critically important. The purpose of this study was to develop a nomogram model to predict long-term risk of CALs one year later.

**Materials and methods:**

This retrospective study analyzed clinical data, laboratory test results, echocardiographic findings, and follow-up information from 2,481 pediatric patients diagnosed with KD who were admitted to the Children’s Hospital of Soochow University between July 2016 and December 2022. Multivariate logistic regression was used to identify factors associated with long-term CAL risk and construct a nomogram. The model’s performance was evaluated using receiver operating characteristic (ROC) curves, areas under the curve (AUC), calibration curves, and decision curve analysis (DCA).

**Results:**

Logistic regression analysis revealed that male sex, prolonged hospitalization, prolonged fever, decreased hemoglobin (Hb), decreased hematocrit (HCT), and hyponatremia were significant predictors of long-term CAL risk in KD patients. In the training dataset, the model achieved an AUC of 0.801, with a sensitivity of 82.5% and a specificity of 65.5%. In the validation dataset, the AUC was 0.796, with a sensitivity of 66.7% and a specificity of 83.5%. The calibration curve was aligned with the predicted curve. Additionally, DCA revealed a high net benefit of the model.

**Conclusion:**

The nomogram prediction model exhibited high accuracy and can help physicians identify KD patients who may have long-term CALs.

## 1 Introduction

Kawasaki disease (KD), also known as mucocutaneous lymph node syndrome, occurs in children aged 6 months to 5 years. The risk of KD in males is up to one and a half times greater than that in females (1). Coronary artery lesions (CALs) are serious complications of KD (2). Previous studies have demonstrated that the probability of KD and CALs co-occurrence is 15%–20%, with CALs manifesting mainly as coronary artery dilatation, coronary artery aneurysm (CAA), giant CAA, coronary stenosis, and myocardial infarction (3).

Kawasaki disease (KD) has replaced rheumatic fever as the most common cause of acquired heart disease in children and is now recognized as a major risk factor for ischemic heart disease in adulthood (4). Coronary artery dilatation is reported to resolve within 4–8 weeks in most cases, but can progress to coronary artery aneurysms (CAAs) in a small subset of patients. Medium to large CAAs, especially giant CAAs, are less likely to regress and may persist for extended periods. About 20%–50% of pediatric patients with such aneurysms develop vascular obstruction, increasing the risk of myocardial ischemia, myocardial infarction, arrhythmia, or sudden death (5–7).

Various authors, including Kobayashi (8), Sato (9), and Tremoulet (10), from different countries and regions have developed several efficacy-sensitive scoring systems on the basis of local KD and CAL data. Although these scoring systems have good predictive efficacy in local regions, they exhibit average efficacy in nonlocal regions. Current CAA scoring methods in China can predict the incidence of CAA in patients with acute KD. However, a predictive model for long-term CAL risk is currently not available (11). This study aimed to comprehensively evaluate the clinical characteristics of patients with KD before they received intravenous immunoglobulin (IVIG) treatment and to establish a long-term prediction model for the occurrence of coronary artery lesions in children with KD in the Suzhou region one year later.

## 2 Materials and methods

### 2.1 Patients

In this study, medical records were retrieved from the electronic medical records system of the Children's Hospital of Soochow University. A total of 2,720 children were diagnosed with Kawasaki disease (KD) between July 2016 and December 2022. The exclusion criteria were as follows: patients with cardiac insufficiency, cardiomyopathy, organic heart disease, autoimmune diseases, skin diseases, hematological diseases, tumors, and other major organ disorders; patients with severe infections; patients without echocardiographic data; and patients with recurrent KD who were not treated with IVIG for personal reasons. After 7 patients who refused intravenous immunoglobulin treatment for personal reasons during hospitalization, 5 cases with recurrent KD, 26 children with prior medical treatment at other hospitals before admission, and 201 cases with severely incomplete clinical data were excluded; a final total of 2,481 patients were included.

Missing data were handled using multiple imputation in SPSS 23.0. Variables with more than 20% missing values, such as CKMB and CTnT, missing long-term coronary outcomes, and loss to follow-up in some patients transferred from other medical centers, were excluded. All included cases met the diagnostic criteria and had complete clinical data, laboratory findings, and follow-up echocardiographic results.

**Figure 1:**
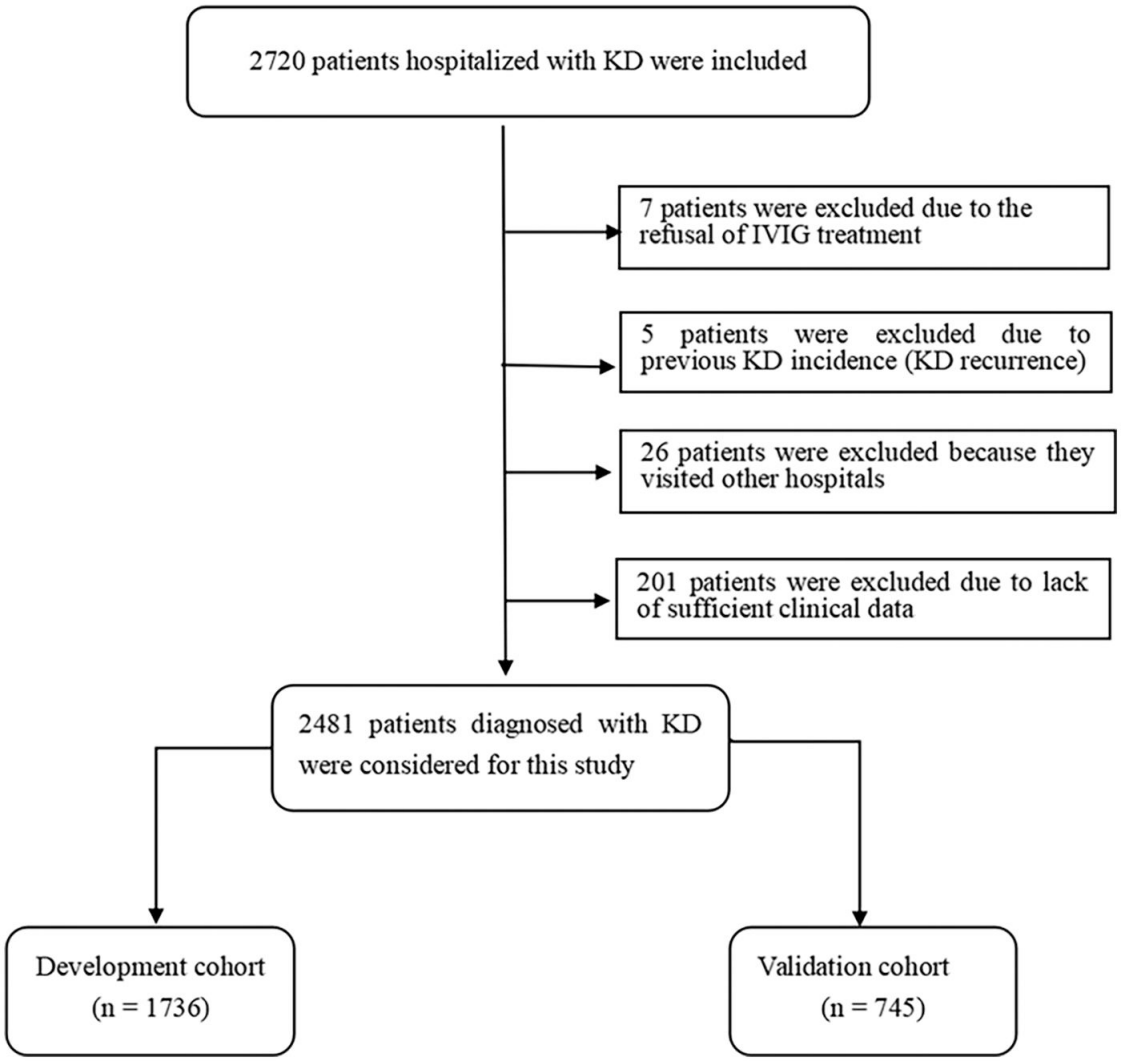
Study flow diagram. KD, Kawasaki disease; IVIG, intravenous immunoglobulin.

The data of 2,481 children with a primary diagnosis of cutaneous mucocutaneous lymph node syndrome who were admitted to the Children's Hospital of Soochow University between July 2016 and December 2022 were retrospectively analyzed. According to the random allocation principle of 7:3, 70% and 30% of patients were divided into the training set and the testing set, respectively. Finally, the data of 1,736 and 745 patients were included in the development cohort (DC) and validation cohort (VC), respectively (Figure 1).

The Children’s Hospital of Soochow University’s institutional review board approved this study (No: 2023cs109).

### 2.2 Definitions

The diagnosis of KD and completed KD (cKD) was based on the 2017 American Heart Association guidelines (4). Z scores for the left main coronary artery, left circumflex artery, left descending artery, and right coronary artery were determined based on previously described methods. CAA was diagnosed when the Z score was ≥ 2.5. CALs were classified based on the following criteria: no involvement, Z score < 2; coronary artery dilation, 2 ≤ Z score < 2.5; small aneurysm, 2.5 ≤ Z score < 5; medium aneurysm, 5 ≤ Z score < 10; and giant aneurysm, Z score ≥ 10. IVIG resistance was defined as persistent fever 36 h after standard treatment or recurrent post-defervescence fever requiring a second IVIG dose or adjuvant therapy.

### 2.3 Data collection

Medical data, including general clinical information, treatment, incidence of IVIG resistance, fever duration before IVIG treatment (hereinafter referred to as: Fever duration), Laboratory test results before IVIG treatment, and follow-up echocardiographic data within 1 year, were collected.

### 2.4 Statistical analyses

The available data were used to determine the sample size. Categorical variables are presented as numbers and percentages, while continuous variables are expressed as medians with interquartile ranges or means ± standard deviations. Comparisons between the two groups were performed using the Mann–Whitney U test, Student's t-test, or chi-square test, as appropriate. Multivariate stepwise logistic regression analysis and Multicollinearity test were conducted to identify potential predictors and construct a nomogram for risk prediction. Calibration plots, decision curve analysis (DCA), and the area under the receiving operating characteristic (ROC) curve (AUC) were used to assess the model's performance.

## 3 Results

### 3.1 Patient characteristics

A total of 2,481 KD patients who were randomly allocated to a DC (*n* = 1736) or a VC (*n* = 745) were included in our study. The detailed demographic and clinical characteristics of the patients are shown in Table 1. The DC consisted of 1,022 (58.87%) males and 714 (41.13%) females with KD; the remaining 453 (60.81%) males and 292 (39.19%) females formed the VC. The majority of patients in both cohorts presented with complete Kawasaki disease (>85%), and at least 90% of the patients were responsive to intravenous immunoglobulin. The white blood cell (WBCs) (*P* = 0.038) and Na^+^ (*P* = 0.035) counts significantly differed between the DC and VC groups. The other indices did not significantly differ between the two groups (*P* = 0.127–0.995). The detailed demographic and clinical characteristics of the patients are shown in Table 1. Thus, all the index data in the study cohort can be used for 2,481 patients.

**Table 1:** Comparative analysis of the clinical features and laboratory parameters of the development and validation cohorts.

Variable	Development cohort (*n* = 1736)	Validation cohort (*n* = 745)	Z/*χ*^2^	*P*
Sex, *n* (%)			χ^2^ = 0.809	0.368
Female	714 (41.13)	292 (39.19)		
Male	1022 (58.87)	453 (60.81)		
Use of IVIG, *n* (%)			χ^2^ = 0.955	0.328
IVIG sensitivity	1622 (93.43)	688 (92.35)		
IVIG insensitivity	114 (6.57)	57 (7.65)		
KD type, *n* (%)			χ^2^ = 0.782	0.377
iKD	213 (12.27)	101 (13.56)		
cKD	1523 (87.73)	644 (86.44)		
Hospital stay duration(days)	10.00 (8.00–12.00)	10.00 (8.00–12.00)	Z = 0.388	0.701
Fever duration(days)	6.00 (5.00–8.00)	6.00 (5.00–8.00)	Z = 1.516	0.136
Age (months)	23.00 (13.00–40.00)	22.00 (13.00–41.00)	Z = 0.481	0.631
Height (cm)	86.25 (76.00–100.00)	85.00 (76.00–100.00)	Z = 0.141	0.888
Weight (kg)	12.00 (10.00–15.43)	12.00 (10.00–15.00)	Z = 0.593	0.554
CRP (mg/L)	65.78 (38.48–104.07)	67.50 (39.15–111.01)	Z = 1.041	0.298
ESR (mm/h)	36.00 (22.00–53.25)	36.00 (21.00–52.00)	Z = 0.626	0.531
WBC (×10^9^ cells/L)	14.58 (11.43–18.49)	13.99 (11.23–18.17)	Z = 2.077	0.038
NE (%)	66.70 (55.48–77.50)	65.50 (53.50–77.60)	Z = 0.973	0.331
LY (%) NE (×10^9^ cells/L)	23.70 (15.28–32.60) 9.65 (6.87–13.45)	23.70 (15.30–33.30) 9.25 (6.63–13.10)	Z = 0.085 Z = 1.120	0.932
				0.263
LY (×10^9^ cells/L)	3.28 (2.13–4.87)	3.18 (2.06–4.71)	Z = 1.201	0.230
NLR	2.90 (1.76–5.43)	2.89 (1.63–5.36)	Z = 0.105	0.916
MO (×10^9^ cells/L)	0.66 (0.44–0.97)	0.64 (0.44–0.96)	Z = 1.237	0.216
EO (×10^9^ cells/L)	0.24 (0.10–0.50)	0.25 (0.10–0.53)	Z = 0.842	0.400
Hb (g/L)	111.50 (105.00–118.00)	111.00 (105.00–117.00)	Z = 0.738	0.461
RDW (%)	13.10 (12.50–14.03)	13.10 (12.50–14.10)	Z = 0.768	0.443
HCT (L/L)	0.34 (0.32–0.37)	0.34 (0.32–0.36)	Z = 1.294	0.196
PLT (×10^9^ cells/L)	368.00 (281.00–496.00)	361.00 (273.00–484.00)	Z = 0.999	0.318
PDW (%)	11.40 (9.70–15.50)	11.30 (9.70–15.40)	Z = 0.551	0.582
Fib (g/L)	5.83 (4.68–7.32)	5.92 (4.70–7.14)	Z = 0.073	0.942
ALB (g/L)	38.55 (34.18–41.20)	38.90 (34.10–41.40)	Z = 0.970	0.332
FAR	0.15 (0.12–0.20)	0.15 (0.12–0.19)	Z = 0.127	0.899
PA (mg/L)	106.00 (77.00–168.25)	103.00 (74.00–166.00)	Z = 1.392	0.164
TBIL (µmol/L)	5.75 (4.00–8.90)	5.90 (4.00–9.20)	Z = 0.554	0.580
TG (mmol/L)	1.30 (1.02–1.72)	1.28 (0.99–1.67)	Z = 1.071	0.284
Triglyceride (mmol/L)	3.57 (3.08–4.05)	3.52 (3.03–3.99)	Z = 1.506	0.132
ALT (U/L)	27.60 (16.38–54.18)	27.80 (15.70–56.90)	Z = 0.209	0.834
AST (U/L)	31.20 (22.30–49.65)	30.60 (22.20–50.50)	Z = 0.007	0.995
D-Dimer (µg/L)	1080.00 (670.00–1880.00)	1130.00 (710.00–1880.00)	Z = 1.115	0.265
Na^+^ (mmol/L)	135.00 (133.00–137.00)	135.00 (133.00–137.00)	Z = 2.123	0.035
K^+^ (mmol/L)	4.00 (3.60–4.30)	4.00 (3.60–4.30)	Z = 0.061	0.952
Ca^2+^ (mmol/L)	1.08 (1.03–1.11)	1.07 (1.03–1.11)	Z = 0.762	0.447
CKMB (ng/mL)	1.30 (0.80–2.40)	1.40 (0.90–2.20)	Z = 0.177	0.860
CTnT (pg/mL)	0.18 (0.04–5.21)	0.09 (0.04–5.01)	Z = 1.006	0.315
CD48^+^	1.90 (1.40–2.60)	1.90 (1.40–2.60)	Z = 0.308	0.758
CD3^–^CD19^+^	28.60 (21.70–36.40)	27.50 (20.35–36.60)	Z = 1.524	0.127

IVIG, intravenous immunoglobulin; KD, Kawasaki disease; iKD, incomplete KD; cKD, complete KD;Fever duration, fever duration before IVIG treatment;CRP, C-reactive protein; ESR, erythrocyte sedimentation rate; WBC, white blood cell; NE, neutrophils; LY, lymphocytes; NLR, NE to LY ratio; MO, monocytes; EO, eosinophils; Hb, hemoglobin; RDW, red blood cell distribution width; HCT, hematocrit; PLT, platelets; PDW, PLT distribution width; Fib, fibrinogen; ALB, albumin; FAR, Fib to ALB ratio; PA, prealbumin; TBIL, total bilirubin; TG, triacylglycerol; ALT, alanine transaminase; AST, aspartate transaminase; CKMB, creatinine kinase MB; CTnT, cardiac troponin T.

### 3.2 Predictive factor screening and prediction model development

**Figure 2:**
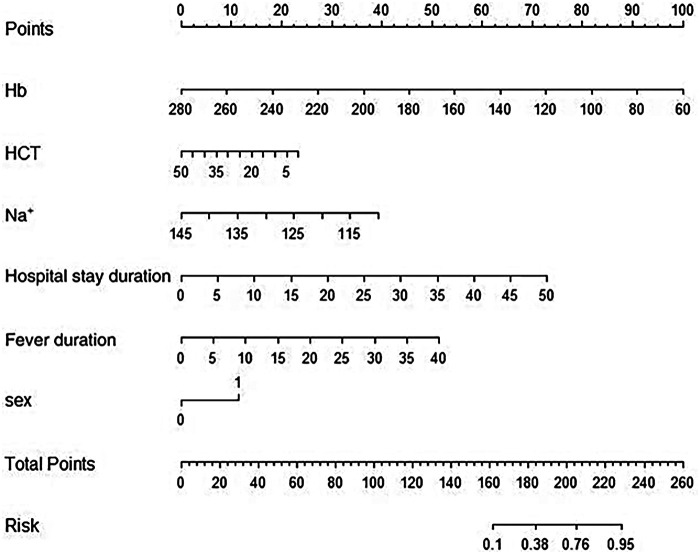
Nomogram for the prediction of long-term coronary artery aneurysm (CAA) risk. Sex scores: The scores for females and males are 0 and 1, respectively. Risk scores: These scores represent the probability that the model can predict the risk of Kawasaki disease with long-term coronary artery lesions.

Univariate logistic regression analysis (Table 2) identified sex, IVIG use, hospital stay duration, fever duration, C-reactive protein (CRP), hemoglobin (Hb), hematocrit (HCT), albumin (ALB), triacylglycerol (TC), and sodium (Na⁺) as significant factors associated with long-term CALs risk. Based on multivariate regression analysis (Table 3) and clinical significance, sex, hospital stay duration, fever duration, Hb, HCT, and Na + were independent high-risk factors for long-term CALs complications in KD patients, and the Multicollinearity test (Table 4) indicates that there is no significant multicollinearity problem among the variables included in this study. These six predictors were incorporated into the nomogram model (Figure 2). A higher overall risk score for each patient corresponds to a greater long-term risk of CALs.

**Table 2:** Univariate logistic regression analysis of potential risk factors.

Variable	OR	95%CI	*P* value
Sex			
Male	1.00	Reference	
Female	0.40	0.22–0.72	0.003*
Use of IVIG, *n* (%)			
IVIG sensitivity	1.00	Reference	
IVIG insensitivity	2.49	1.20–5.18	0.015*
KD type			
cKD	1.00	Reference	
iKD	1.72	0.90–3.29	0.099
Hospital stay duration(days)	1.15	1.10–1.19	< 0.001*
Fever duration(days)	1.14	1.06–1.23	< 0.001*
Age (months)	1.00	0.98–1.01	0.439
Height (cm)	0.99	0.98–1.01	0.292
Weight (kg)	1.01	0.97–1.05	0.524
CRP (mg/L)	1.01	1.01–1.01	0.002*
ESR (mm/h)	1.01	1.00–1.02	0.197
WBC (×109/L)	1.03	0.99–1.07	0.193
NE (%)	0.99	0.98–1.00	0.090
LY (%)	1.01	0.99–1.02	0.357
NE (×10^9^ cells/L)	1.00	0.97–1.02	0.853
LY (×10^9^ cells/L)	1.00	0.98–1.02	0.982
NLR	0.99	0.94–1.03	0.569
MO (×10^9^ cells/L)	1.10	0.97–1.24	0.153
EO (×10^9^ cells/L)	1.27	0.77–2.11	0.345
Hb (g/L)	0.95	0.92–0.97	< 0.001*
RDW (%) HCT (L/L)	0.99 0.95	0.94–1.05 0.91–0.99	0.768 0.039*
PLT (×10^9^ cells/L)	1.00	1.00–1.00	0.551
PDW (%)	0.94	0.86–1.02	0.156
Fib (g/L)	0.99	0.97–1.01	0.552
ALB (g/L)	0.98	0.96–0.99	0.004*
FAR	1.00	0.91–1.10	0.928
PA (mg/L)	1.00	0.99–1.00	0.437
TBIL (µmol/L)	1.00	0.98–1.02	0.730
TG (mmol/L)	1.08	0.99–1.18	0.103
Triglyceride (mmol/L)	0.69	0.50–0.94	0.020*
ALT (U/L)	1.00	1.00–1.00	0.856
AST (U/L)	1.00	1.00–1.00	0.632
D-Dimer (µg/L)	1.00	1.00–1.00	0.904
Na^+^ (mmol/L)	0.89	0.86–0.92	< 0.001*
K^+^ (mmol/L)	1.04	0.95–1.14	0.368
Ca^2+^ (mmol/L)	0.98	0.75–1.29	0.887
CKMB (ng/mL)	1.00	0.97–1.03	0.848
CTnT (pg/mL)	0.99	0.94–1.04	0.713
CD48^+^	0.98	0.84–1.15	0.803
CD3^–^CD19^+^	1.00	0.98–1.02	0.925

OR, odds ratio; CI, confidence interval; IVIG, intravenous immunoglobulin; KD, Kawasaki disease; iKD, incomplete KD; cKD, complete KD; Fever duration, fever duration before IVIG treatment; CRP, C-reactive protein; ESR, erythrocyte sedimentation rate; WBC, white blood cell; NE, neutrophil; LY, lymphocyte; NLR, NE-to-LY ratio; MO, monocyte; EO, eosinophil; Hb, hemoglobin; RDW, red blood cell distribution width; HCT, hematocrit; PLT, platelet; PDW, PLT distribution width; Fib, fibrinogen; ALB, albumin; FAR, Fib-to-ALB ratio; PA, prealbumin; TBIL, total bilirubin; TG, triacylglycerol; ALT, alanine transaminase; AST, aspartate transaminase; CKMB, creatinine kinase MB; CTnT, cardiac troponin T.

* *P* < 0.05.

**Table 3:** Results of the multivariate logistic regression analysis.

Variable	*β*	OR	95%CI	*P* value
Sex				
male		1.00	Reference	
female	−0.88	0.42	0.22–0.79	0.007
Hospital stay duration(days)	0.11	1.12	1.07–1.17	< 0.001
Fever duration(days)	0.10	1.10	1.02–1.19	0.011
Hb (g/L)	−0.03	0.97	0.94–0.99	0.005
HCT (L/L)	−0.04	0.96	0.92–1.01	0.126
Na^+^ (mmol/L)	−0.09	0.92	0.89–0.95	< 0.001

OR, odds ratio; CI, confidence interval; Hb, hemoglobin; HCT, hematocrit;Fever duration, fever duration before IVIG treatment.

**Table 4:** Multicollinearity test.

Variable	VIF	1/VIF
Sex	1.01	0.99
Hospital stay duration(days)	1.016	0.985
Fever duration(days)	1.036	0.966
Hb (g/L)	1.022	0.979
HCT (L/L)	1.021	0.98
Na^+^ (mmol/L)	1.042	0.959

Hb, hemoglobin; HCT, hematocrit; Fever duration, fever duration before IVIG treatment.

### 3.3 Accuracy and net benefit of the model

**Figure 3:**
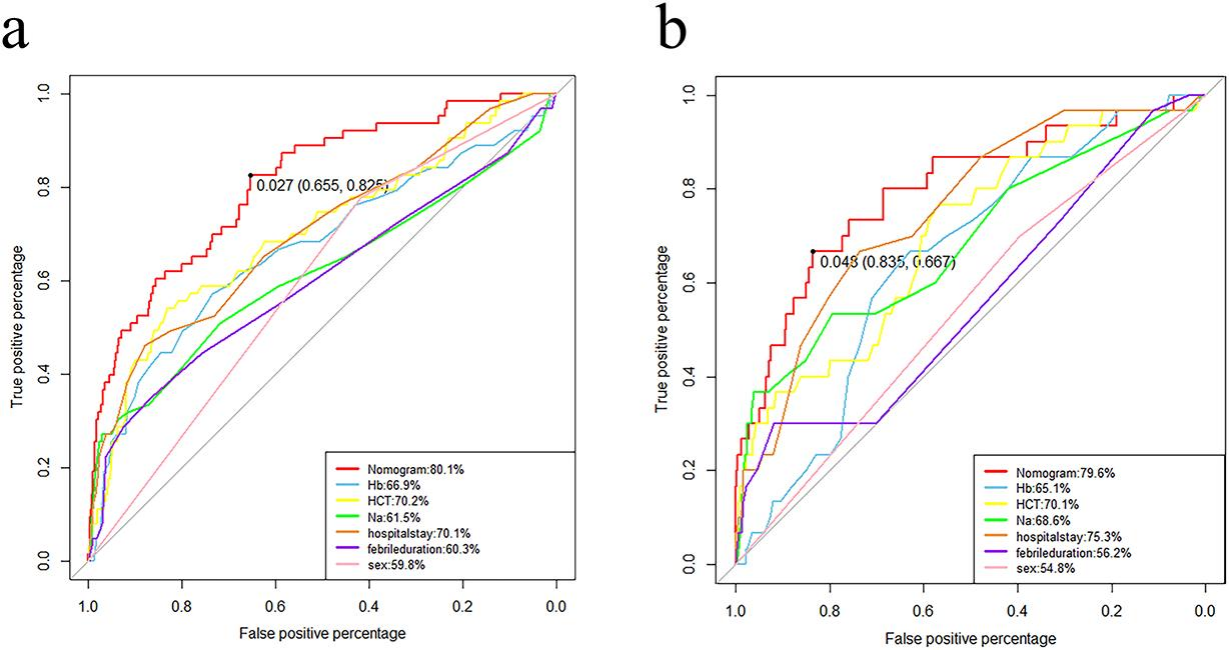
Receiver operating characteristic (ROC) curves of the prediction model. **(a)** Development cohort **(b)** Validation cohort.

**Figure 4:**
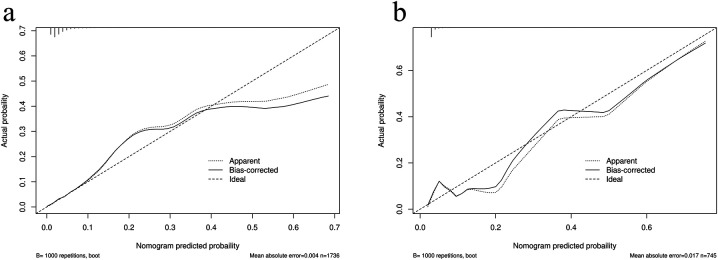
Calibration curve for the prediction model. **(a)** Development cohort **(b)** Validation cohort.

**Figure 5:**
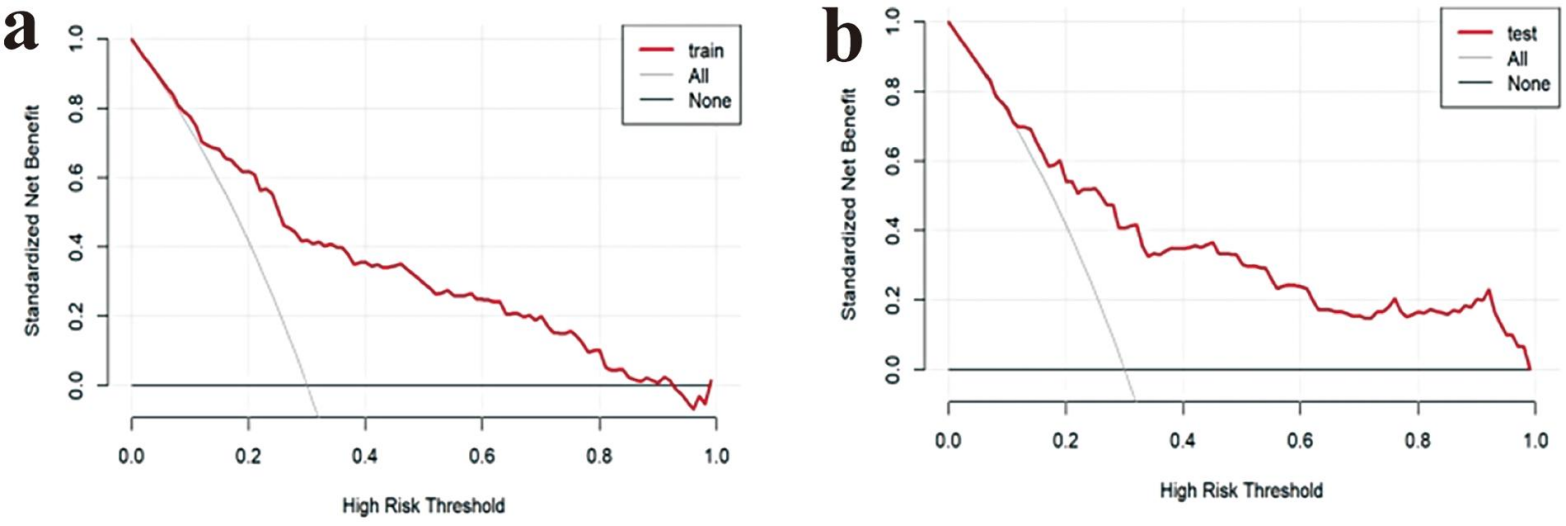
Decision curve analysis of the prediction model. **(a)** Development cohort **(b)** Validation cohort.

The AUC, sensitivity, and specificity of the model in the DC group were 0.801, 82.5%, and 65.5%, respectively (Figure 3A). The calibration curve aligned with the predictive curve (Figure 4A). DCA revealed a high net benefit of the predictive model (Figure 5A). Internal validation was performed with the VC group. The AUC, sensitivity, and specificity of the model in the VC group were 0.796, 66.7%, and 83.5%, respectively (Figure 3B). Additionally, the model exhibited good consistency. Furthermore, the VC group's calibration curve aligned with the predicted curve (Figure 4B). DCA revealed a significant net benefit of the model in the VC group (Figure 5B).

## 4 Discussion

In this study, a model for predicting long-term CAL risk in KD patients was developed. The prediction model, developed using parameters such as sex, hospital stay duration, fever duration, Hb level, HCT, and Na^+^, performed admirably in both the DC and VC groups. Patients were randomly divided into DC and VC,we consider that the differences in Na^+^ and WBC were random variations caused by sample splitting. Although there was a significant difference in sodium ion concentration between the DC and the VC,After Na^+^ was incorporated into the nomogram model, the AUC of the DC model was 0.801, with a sensitivity of 0.825 and a specificity of 0.655. Another VC was used for external validation. The AUC was 0.796, with a sensitivity of 0.667 and a specificity of 0.835. The discriminative performance was highly consistent between the training and validation sets, suggesting no significant overfitting of the model. As a predictor, serum sodium levels demonstrate robust clinical applicability. Some discrepancies in sensitivity and specificity were observed between the DC and VC. The sensitivity of the DC was relatively higher, but the specificity was lower, whereas the sensitivity and specificity of the VC were slightly lower and higher, respectively. These variations were attributed mainly to minor shifts in the optimal cut-off value across populations. Notably, the AUC values were highly consistent between the two cohorts, demonstrating the robust and stable discriminatory ability of the model. Additionally, no significant overfitting was detected, further confirming the favorable generalizability of the established prediction model.

The incidence of CALs was associated with male sex in pediatric patients with KD, which is consistent with the results of a previous study (12). Zhang et al. reported that male children were more prone to coronary artery damage than female children were. The authors reported that the average body mass index (BMI) of male children in this study was significantly greater than that of female children. Thus, BMI may be an important factor for determining the correlation between sex and CALs, although these findings must be further verified in future studies.

Since our objective was to predict the one-year risk of CALs in patients with KD, we therefore incorporated the Hospital stay duration stay into this prediction model. In this study, hospital stay duration was a risk factor for CAL complications among KD patients (odds ratio (OR) = 1.12; 95% confidence interval (CI) = 1.07–1.17; *P* < 0.001). This can be attributed to delayed IVIG treatment or IVIG treatment insensitivity due to late diagnosis, which increases the risk of developing CALs. A previous study (12) reported that the probabilities of CALs in the late IVIG and early IVIG treatment groups were 27% and 1%, respectively. Sano et al. (13) reported that the incidence of CALs in children without an IVIG response was greater than that in children with an IVIG response (71% vs. 5%, *P* < 0.0001). Therefore, clinicians must detect and diagnose KD early in children to enable standardized treatment initiation, decrease the duration of hospital stay, and reduce the risk of CALs.

In pediatric patients with KD, fever is the most common clinical manifestation. Several studies (14, 15) have reported that fever is an independent risk factor for KD complicated by CALs. Ilaria Maccora (16) performed long-term follow-up studies (1–36 years) and reported that the duration of fever before IVIG treatment was directly proportional to the risk of the development of CALs in patients with KD, which is consistent with the findings of this study. This association may be attributed to the increased risk of cardiovascular endothelial injury in pediatric KD patients due to the induction of inflammatory factors (17). In future studies, we will strive to minimize subjective bias during the collection of patients’ medical histories and eliminate data source heterogeneity arising from different calculation methods, thereby avoiding potential inconsistencies in research conclusions induced by these confounding factors.

Inflammation tends to be prolonged in pediatric patients with clinical anemia. In this study, hypohemoglobinemia was identified as a high-risk factor for the development of CALs. However, the underlying mechanism remains unclear. We hypothesize that hypohemoglobinemia in pediatric KD patients is closely linked to hepcidin, which can directly impair the proliferation of red blood cell precursors and inhibit the formation and survival of erythroid colonies (18). Kuo HC et al. (19) reported that changes in hepcidin levels were associated with the development of KD complicated by CALs. The pathophysiological relationship between hypohemoglobinemia and CALs should be further investigated through large-scale, multicenter studies.

In this study, hematocrit (HCT) was incorporated into the model using R software based on multivariate stepwise logistic regression analysis and clinical significance. The HCT is an important factor in the oxygen supply and determines blood viscosity. In adults, HCT is significantly and negatively correlated with coronary artery disease (20); furthermore, hematocrit levels tend to decrease as the severity of arterial stenosis increases. Epidemiological studies in Japan have revealed that the incidence of CALs in children with low HCT is about 0.45 times greater than that in healthy children. In the present study, a low HCT was a risk factor for CALs. To date, however, the mechanism underlying the occurrence of hypohematocrit in KD children complicated with CALs remains unclear. Related studies have shown that anemia in KD children is thought to prolong the duration of active inflammation (22). Specifically, inflammatory cytokines can alter the production of erythropoietin, reduce the rate of bone marrow hematopoiesis, and disrupt iron metabolism processes, thereby impairing erythrocyte production and maturation, an effect that is also considered the primary cause of anemia in KD children. Although the aforementioned studies revealed an association between low HCT and CALs, pediatric hematopoietic function significantly changes with age and physical development, resulting in distinct reference ranges for hematological parameters. Consequently, clearly elucidating the relationship between these two factors remains a considerable challenge. Further in-depth investigations, with adjustments for age and physiological developmental differences, are warranted to better clarify this correlation. Therefore, this study strictly followed the guidelines for the construction of clinical prediction models. HCT has important clinical guiding significance and helps improve the overall predictive performance of the model; thus, it was retained even when *P* > 0.05 in the multivariate analysis to avoid model selection bias and the lack of clinical rationality caused by selecting variables solely based on statistical significance as much as possible.

A retrospective analysis by Suzuki H et al. (21) revealed that sodium levels in pediatric patients with CALs were significantly lower than those in patients without CALs. This downregulation was observed only during the second week after disease onset. Therefore, progressive interstitial tissue edema within two weeks of onset may represent an important risk factor for CALs. Additionally, decreased sodium levels were identified as a risk factor in pediatric patients with KD complicated by CALs. However, this study assessed sodium levels only before IVIG treatment during the acute stage and did not monitor subsequent changes dynamically. As a result, the impact of sodium downregulation on the development of CALs remains unclear. In this retrospective observational study, hyponatremia should be recognized as a predictive biomarker rather than a modifiable therapeutic target. Our results demonstrate a significant association but do not establish causality, nor do they provide evidence that early correction of sodium imbalance reduces the long-term risk of CALs. Future prospective cohort studies or randomized controlled trials are warranted to dynamically characterize serum sodium fluctuations during the acute phase of KD, elucidate the pathophysiological mechanisms linking electrolyte disorders to coronary injury, and validate whether prompt monitoring and correction of water–electrolyte imbalance in the acute stage can improve long-term coronary outcomes. These investigations will facilitate the translation of observational risk markers into clinically actionable strategies.

This study has several limitations. In this study, we constructed the prediction model using univariate screening followed by stepwise multivariate logistic regression. While this approach is straightforward and widely applied, it has inherent limitations. Stepwise selection may lead to unstable variable selection and increase the risk of model overfitting. Although we performed random split validation and collinearity diagnostics, our model still carries a potential risk of bias. These methodological drawbacks may somewhat limit the generalizability of our findings. Future studies are warranted to adopt more robust algorithms such as LASSO regression, coupled with bootstrap resampling for internal validation, to further enhance model stability and reliability. As this was a retrospective, single-center study, it is susceptible to data bias. Moreover, echocardiographic findings may be influenced by selection bias due to equipment variability or subjective interpretation.The patients excluded from the study and those included in the cohort may have differed systematically, which could also lead to selection bias. Therefore, the predictive value of this model for KD patients with CALs should be validated through multicenter, prospective studies with larger sample sizes.

## 5 Conclusions

This retrospective study revealed that male sex, prolonged hospitalization, prolonged fever duration, decreased Hb level, reduced HCT, and hyponatremia are high-risk factors for long-term CAL complications in KD patients. The prediction model for long-term CAA risk after KD onset exhibited excellent performance. Therefore, pediatricians must carefully monitor these factors in the clinic.

## Data Availability

The raw data supporting the conclusions of this article will be made available by the authors, without undue reservation.
